# *Enterococcus durans* EP1 a Promising Anti-inflammatory Probiotic Able to Stimulate sIgA and to Increase *Faecalibacterium prausnitzii* Abundance

**DOI:** 10.3389/fimmu.2017.00088

**Published:** 2017-02-10

**Authors:** Paula Carasi, Silvia María Racedo, Claudine Jacquot, Anne Marie Elie, María de los Ángeles Serradell, María C. Urdaci

**Affiliations:** ^1^UMR 5248, Laboratoire de Microbiologie et Biochimie Appliquée (LBMA), Bordeaux Sciences Agro, Université de Bordeaux, Gradignan, France; ^2^Cátedra de Microbiología, Facultad de Ciencias Exactas, Departamento de Ciencias Biológicas, Universidad Nacional de La Plata (UNLP), La Plata, Argentina; ^3^CCT-La Plata, CONICET, La Plata, Argentina

**Keywords:** *Enterococcus durans*, probiotic, IgA, *Faecalibacterium prausnitzii*, anti-inflammatory

## Abstract

*Enterococcus* species, principally *Enterococcus faecium* are used as probiotics since a long time with preference in animal applications but safety considerations were updated and also new uses as probiotics can be envisaged. Fifteen *Enterococcus* strains isolated from different foods were identified and analyzed for virulence factors and antibiotic resistance. Three *Enterococcus durans* strains were selected to study their immunomodulatory properties on PBMC and Caco2 cells. Two strains presented a profile toward a mild inflammatory Th1 response considering TNF-α/IL-10 and IL-1β/IL-10 cytokines ratios. The third strain EP1, presented an anti-inflammatory potential and was selected for *in vivo* studies. In mice, the strain was well tolerated and did not cause any adverse effects. EP1 administration increased the amount of IgA+ cells in mesenteric lymph node (MLN) after 7 days of administration. In fecal samples, the IgA content increased gradually and significantly from day 7 to day 21 in treated group. Additionally, IL-17, IL-6, IL-1β, IFN-γ, and CXCL1 gene expression significantly decreased on day 21 in Peyer’s patches and IL-17 decreased in MLN. Mice treated with the probiotic showed significant lower mRNA levels of pro-inflammatory cytokines and mucins in the ileum at day 7 while their expression was normalized at day 21. Colonic expression of il-1β, il6, and mucins remain diminished at day 21. Ileum and colon explants from treated mice stimulated *in vitro* with LPS showed a significant reduction in IL-6 and an increase in IL-10 secretion suggesting an *in vivo* protective effect of the probiotic treatment against a proinflammatory stimulus. Interestingly, analysis of feces microbiota demonstrated that EP1 administration increase the amount of *Faecalibacterium prausnitzii*, a butyrate-producing bacteria, which is known for its anti-inflammatory effects. In conclusion, we demonstrated that EP1 strain is a strong sIgA inducer and possess mucosal anti-inflammatory properties. This strain also modulates gut microbiota increasing *Faecalibacterium prausnitzii*, a functionally important bacterium. Thus, *E. durans* EP1 is not only a good candidate to increases *F. prausnitzii* in some cases of dysbiosis but can also be interesting in gut inflammatory disorders therapy.

## Introduction

Enterococci are an ancient genus of lactic acid bacteria (LAB) that are highly adapted to living in complex environments and surviving adverse conditions. They are ubiquitous, inhabiting the gastrointestinal tracts of a wide variety of animals, from insects to man. This widespread pattern of colonization suggests that enterococci have been members of the gut microbiome of ancient common ancestors (1). Enterococcal strains can be found in a variety of fermented foods contributing to the ripening and aroma development of certain cheeses or fermented sausages, as well as probiotics to improve human or animal health (2, 3). However, the genus *Enterococcus* is a controversial group of LAB considering that some strains may be associated with human infections (4–6). Virulence and pathogenicity factors such as adhesins, invasins, pili, and hemolysin have been described principally on *Enterococcus faecalis* and *Enterococcus faecium*, but other enterococcal species occasionally can cause human infections (7). Trivedi et al. (8) showed the presence of virulence genes in other enterococcal species isolates from foods. Antibiotic-resistant enterococci are widespread in food including dairy and meat products and can be a potential reservoir of antibiotic resistance gene exchanges between enterococci and other species of bacteria (2, 9). Therefore, susceptibility to clinically relevant antibiotics of *Enterococcus* strains isolated from food stuffs is very important for consumer health.

*Enterococcus faecium* is one common species used as probiotic in animal feed (10) and concerning its safety, the European Food Safety Authority (EFSA) edited a new guidance document (11) to differentiate between safe and potentially harmful clinical strains, based in their susceptibility to ampicillin and the absence of three genetic markers associated with virulence (*esp, hylEfm, IS16*). In animals, enterococcal probiotics are mainly used to treat or prevent diarrhea, for immune stimulation or to improve growth. For example, *E. faecium* reduced the portion of piglets suffering diarrhea and improved their performances (12) or reduced the intestinal colonization by enteropathogenic bacteria (13). *E. faecium* SF68^®^ (NCIMB 10415) approved for use as feed additive for different animal productions (14) reduced the pathogenic bacterial load in animals declining the virulence gene expression of the resident *Escherichia coli* and conferred an anti-inflammatory response (15). Further, SF68 strain has been reported to possess immune stimulatory effect on dogs (16).

Most of human probiotics consist of *Lactobacillus* spp. and *Bifidobacterium* spp., whereas less information exists about the effectiveness of enterococcal strains as probiotics. In humans, *Enterococcus* strains have been used for treatment of diseases such as diarrhea or antibiotic associated diarrhea, inflammatory pathologies that affects colon such as irritable bowel syndrome (IBS), or immune regulation (17). *E. faecium* SF68 is specially used for the treatment of diarrhea in children (18) and to prevent diarrhea caused by antibiotic treatments, as demonstrated for example in a multicenter, placebo-controlled double-blinded clinical study (19). Moreover, enterococcal strains have been used for health improvement such as lowering cholesterol levels (20, 21).

Now, probiotics can be considered as a therapeutic option for treatment of allergy and even for inducing or maintaining clinical remission of IBS. *E. faecalis* Symbioflor 1, an immunomodulatoty strain, has been used to combat recurrent, chronic sinusitis or bronchitis and to help to asthma treatment in school children (22, 23). *E. faecium* Paraghurt^®^ has demonstrate its efficacy in lowering the symptoms associated with IBS in a clinical study (24) as well as *E. faecium* PR88 (25) and the multi-strains probiotic ProSymbioflor^®^ (*E. faecalis* and *E. coli*) (26). The probiotic Medilac DS^®^ (*E. faecium* and *Bacillus subtilis*) has shown to decrease the severity and frequency of abdominal pain (27).

Immunomodulatory properties are very important in the mode of action of probiotics. Numerous studies analyze the immunomodulatory power of different species of *Lactobacillus* and *Bifidobacteria in vitro* or eventually *in vivo*. Even though not many researchers have studied the immunomodulatory properties of *Enterococcus* strains, nowadays the interest in this species is increasing. Tarasova et al. (28) described that *E. faecium* L5 was able to restore the microbiota and increase the expression of IL-10 and decrease the IL-8 expression in a rat model of dysbiosis. Further, studies with *E. faecalis* CECT 7121 or *E. faecium* JWS 833 demonstrated their ability to enhance cytokine production on dendritic cells (29, 30).

Avram-Hananel et al. (31) demonstrated *in vitro* and also *in vivo* using a murine model of colitis that *Enterococcus durans* M4-5, a high-butyrate-producing strain induces significant anti-inflammatory effects, mediated by regulation of pro- and anti-inflammatory immune factors inhibiting the development of dextran sodium sulfate (DSS) induced colitis. Similarly, the use of *E. durans* TN-3 alleviates DSS colitis through the induction of Treg cells and the restoration of the diversity of the gut microbiota (32).

In order to select new potentially interesting probiotics, we identified several strains of *Enterococcus* spp. isolated from different sources in order to assess relevant functional and safety aspects including presence of virulence genes and susceptibility to antibiotics. From 15 isolates, we choose 3 *E. durans* strains to test their anti-inflammatory potential *ex vivo*. Finally, one *E. durans* strain was selected to performed studies in healthy mice in order to analyze mucosal immunomodulatory capacities and its ability to modulate intestinal microbiota.

## Materials and Methods

### Bacterial Strains and Growth Conditions

*Enterococcus* strains isolated from different sources were used in this work as well as several collection strains, all of them all listed in Table 1. These bacteria were grown using M17 broth (DIFCO, Detroit, MI, USA) in agitation at 37°C for 24 h.

**Table 1:** ***Enterococcus* strains used in the study, origin, and presence of virulence genes**.

Strain	Origin	Species	acm	GelE	cylA	VanA	VanB	VanC2	Agg	ccf	espfm	IS16	HylEfm
4812	CHU Bordeaux^a^	*E. faecium*	−	+	+	+	−	−	−	−	+	nd	nd
5088	CHU Bordeaux^a^	*E. faecium*	−	+	−	−	−	−	−	−	+	nd	nd
3091	CHU Bordeaux^a^	*E. faecium*	−	−	−	−	−	+	−	−	−	nd	nd
3092	CHU Bordeaux^a^	*E. faecium*	+	−	−	−	−	−	−	−	−	nd	nd
6569	ATCC	*E. faecium*	+	−	−	−	−	−	−	−	−	−	−
29212	ATCC	*E. faecalis*	−	+	+	−	−	−	−	+	−	−	−
51299	ATCC	*E. faecalis*	−	+	−	−	+	−	+	+	−	−	−
25390	DSMZ	*E. faecium*	nd	nd	nd	nd	nd	nd	nd	nd	nd	+	+
5348	CIP	*E. hirae*	−	−	−	−	−	−	−	−	−	nd	nd
EP1	Cow milk^b^	*E. durans*	−	−	−	−	−	−	−	−	−	−	−
EP2	Cow milk^b^	*E. durans*	−	−	−	−	−	−	−	−	−	−	−
EP3	Cow milk^b^	*E. durans*	−	−	−	−	−	−	−	−	−	−	−
109	Chicken intestine	*E. faecium*	+	−	−	−	−	−	−	−	−	−	−
433	Chicken intestine	*E. faecium*	+	−	−	−	−	−	−	−	−	−	−
440	Chicken intestine	*E. faecium*	+	−	−	−	−	−	−	−	−	−	−
537	Chicken intestine	*E. faecium*	+	−	−	−	−	−	−	−	−	−	−
545	Chicken intestine	*E. faecium*	+	−	−	−	−	−	−	−	−	−	−
555	Chicken intestine	*E. hirae*	−	−	−	−	−	−	−	−	−	−	−
68	Probiotic^c^	*E. faecium*	+	−	−	−	−	−	−	−	−	−	−
638	Chicken intestine	*E. faecium*	+	−	−	−	−	−	−	−	−	−	−
1439	Goat cheese	*E. faecium*	+	−	−	−	−	−	−	−	−	−	−
1440	Sheep milk^d^	*E. faecium*	+	−	−	−	−	−	−	−	−	−	−
1442	Sheep milk^d^	*E. faecium*	+	−	−	−	−	−	−	−	−	−	−
1443	Sheep milk^d^	*E. durans*	−	−	−	−	−	−	−	−	−	−	−

*^a^Centre Hospitalo-Universitaire de Bordeaux, France*.

*^b^Cow milk origin Argentina*.

*^c^Spring Valley (USA)*.

*^d^Sheep milk origin Spain*.

*CIP, Collection of Institut Pasteur*.

Other strains used in this work were *S. aureus* ATCC 6538, *Shigella flexneri* ATCC 9199, *Pseudomonas aeruginosa* ATCC 15442, a clinical isolate *Salmonella enterica* serovar Enteritidis CIDCA 101 (Hospital de Pediatría Prof. Juan P. Garrahan, Buenos Aires, Argentina), enterohaemorragic *Escherichia coli* EDL 933, *Bacillus cereus* ATCC 10876, and *Listeria monocytogenes* ATCC 7644. All strains were grown in brain heart infusion broth (BIOKAR) in aerobic conditions at 37°C for 16 h.

### Molecular Identification

Genomic DNA from *Enterococcus* strains was extracted using the Genomic DNA Purification Kit (Fermentas, France) according to manufacturer’s specifications.

Species identification were confirmed by 16S rDNA gene sequencing and species-specific primers based on the superoxide dismutase (sodA) gene (33, 34).

### *In Vitro* Safety Evaluation

#### Detection of Virulence Genes

All isolates were tested for the presence of the three genetic elements considered relevant for EFSA (11): enterococcal surface protein (*esp*), *IS16*, and *hyl_Efm_*. Other virulence genes included in this study were sex pheromones (*ccf*), gelatinase (*gelE*), cytolysin (*cylA*), aggregation substance (*agg*) (35), cell-wall anchored collagen adhesin (*acm*) (36), and *van A, van B, van C2* (37). Positive controls were used in all PCR reactions (Table 1).

#### Antibiotic Susceptibility and Hemolytic Activity

Susceptibility to antibiotics was evaluated as described previously (34). Briefly, the disk diffusion method (38) was used for ciprofloxacin, gentamicin, sulfamethoxazole + trimethoprim, linezolid, and vancomycin. In the case of ampicillin, the minimum inhibitory concentration was determined by broth microdilution according to ISO 20776-1 (39).

Hemolysis was tested by growth of the strains on Columbia agar (bioMérieux, France) supplemented with 5% human blood (group O) and incubated for 48 h at 37°C under aerobic conditions (34).

### Growth Inhibition of Bacterial Pathogens

An agar spot test was performed to assess antimicrobial properties as described previously (34). Inhibition was considered negative if the width of the clear zone around the colonies was less than 2 mm, a low inhibition capacity was considered if the width of the clear zone ranged between 2 and 5 mm, and a high inhibition capacity was considered if the width was 6 mm or larger. Three independent experiments were performed.

### Resistance to Gastrointestinal Tract Conditions and Adhesion to Mucin and Caco-2 Cells

Resistance to simulated gastric and intestinal compartments was assessed as previously described (34). Briefly, bacterial suspensions were incubated sequentially in solutions simulating the gastric and intestinal compartments. Initially, bacteria suspensions were incubated at 37°C with stirring at 200 rpm for 90 min in simulated gastric fluid (in w/v: 0.73% NaCl, 0.05% KCl, 0.4% NaHCO_3_, and 0.3% pepsin) at pH 2.5. Afterward bacteria were resuspended in simulated intestinal fluid (comprising 0.1%, w/v, pancreatin and 0.15%, w/v, bovine bile salts) at pH 8.0 and incubated at 37°C with stirring at 200 rpm for 3 h. Cell viability was assessed by plate counting. Independent experiments were performed at least three times.

Bacterial binding assays were performed using bacteria before and after performing the gastrointestinal tract simulation experiment. Bacterial binding assays to commercial type III porcine gastric mucin (Sigma-Aldrich) were performed as described previously (40) and adhesion to Caco-2 cells were performed following the protocol described by Minnaard et al. (41). Independent experiments were performed at least three times.

### PBMC and Caco-2 Stimulation Experiments

#### PBMC and Caco-2 Preparation and Stimulation

Peripheral blood samples were obtained from healthy blood donors (Regional Blood Transfusion Center, EFS Aquitaine, Bordeaux, France), and all subjects gave written informed consent in accordance with the Declaration of Helsinki. PBMC were isolated on Ficoll hypaque gradients as described previously (42). Caco-2 cells were cultured as described previously (40).

For cells stimulation experiments, bacteria in stationary phase of growth were harvested by centrifugation and washed three times with PBS. Stimulation experiments were performed by coculturing 2 × 10^7^ of bacteria per well (MOI = 10). Culture supernatants were collected after 24 h of culture, and triplicates were kept at −80°C until cytokine analysis. Cells were detached by mechanical scraping in order to check their viability using the protocol (MTT) described by Minnaard et al. (41) or preserved in RNAlater (QIAGEN, Hilden, Germany) for gene expression studies.

#### Quantification of Cytokine Levels in Cell Culture Supernatants

The cytokine profiles were analyzed after *E. durans* strains stimulation of PBMC using the human Th1/Th2 11plex FlowCytomix Kit (eBioscience). It was designed to measure human IFN-γ, IL-1β, IL-2, IL-4, IL-5, IL-6, IL-8, IL-10, IL-12 p70, TNF-α, and TNF-β. Analysis was performed in a flow cytometer BD Accuri C6 (BD Biosciences). TGF-β was measured using the eBioscience human/mouse TGF beta 1 Ready-SET-Go!^®^ ELISA Kit.

#### Quantification of Gene Expression in Caco-2 by qRT-PCR

Total RNA was isolated using the RNeasy Mini Kit (QIAGEN, Hilden, Germany) with an additional DNase treatment (Turbo DNA-free, Ambion, Inc.) according to the manufacturer’s instructions. One microgram of total RNA was reverse transcribed using the Maxima^®^ Reverse Transcriptase (Fermentas, France) with anchored-oligo (dT) 18 primer. Quantitative real-time PCR analyses were performed using a CHROMO 4™ System (Bio-Rad). The reaction mixture comprised Maxima SYBR Green/ROX qPCR Master Mix (Fermentas, France). Target gene copy numbers were normalized against the housekeeping genes hypoxanthine phosphoribosyltransferase and β2 microglobulin (B2m). Genes evaluated: *il1b, il6*, and *il8*.

### *In Vivo* Experiments

#### Mice

Male Swiss albine mice, 5-week-old (Janvier, Le Genest-Saint-Isle, France) were quarantined 2 weeks after arrival and were housed under standard laboratory conditions with free access to food and water. The temperature was kept at 22°C, and a 12-h light/dark schedule was maintained. Mice were divided into two groups (*n* = 12/group) and received by gavage 10^8^ CFU of *E. durans* EP1 (EP group) or PBS (control group) daily for 7 and 21 days; at each time point six mice were sacrificed. This study was carried out in accordance with the recommendations of the European Economic Community (directive 2010/63/UE). The protocol was approved by the Animal Research Committee of the Agriculture Ministry and the Ethical Committee C2EA50.

#### Safety Evaluation

Mice were weighted every 2 days, behavior and signs of pain were analyzed daily.

At the end of the experimental protocol, ileum and colon sections were preserved for histological studies and liver and spleen were removed and blood samples were collected aseptically. Liver and spleen were homogenized in 0.1% sterile PBS (0.1 g of organ per mL), and ileum content was washed with sterile PBS, and serially diluted. The dilutions were plated on violet red bile glucose (VRBG) Agar (Biokard Diagnostic, Beauvais, France) for enterobacteria, De Man, Rogosa and Sharpe (MRS) agar for LAB, and Yeast extract glucose chloramphenicol (YGC) agar for yeasts. Plates were incubated under anaerobic conditions for 24 h at 37°C for VRBG and YGC, and for 48 h at 37°C for MRS, before examination (40).

#### Tissue and Stool Sampling

Stools were collected at days 7, 14, and 21 and stored at −80°C until analysis. At the end of the experimental protocol, days 7 or 21, ileum and colon samples were collected and were preserved at −20°C in RNAlater. On day 21, Peyer’s patches (PP) and mesenteric lymph nodes (MLN) were also removed and preserved at −20°C in RNAlater for expression analysis, and ileum and colon sections were collected in RPMI medium.

#### Reactivity of Ileum and Colon Explants to LPS

Ileum and colon explants were cultured in RPMI 1640 supplemented with 10% bovine fetal serum and antibiotics, in presence or absence of 10 µg/mL of LPS from *E. coli* as proinflammatory stimulus (all from Sigma Chemical Co., St. Louis, MO, USA) for 24 h at 37°C in an atmosphere of 95% air and 5% CO_2_ as described previously (40, 42). Supernatants were collected, centrifuged, and frozen until cytokine (IL-6, IL-4, IL-10, IL-17A, IFN-γ, and GM-CSF) measurements (Ready-SET-Go!^®^ ELISA Kit, eBioscience, France). All assays were performed according to the manufacturer’s instructions.

#### Quantification of Gene Expression in Mouse Ileum, Colon, MLN, and PP Samples by qRT-PCR

The same procedures described in the section “Quantification of Gene Expression in Caco-2 by qRT-PCR” were used. Cytokine and chemokine genes evaluated were *il1b, il6, il10, il12p70, il17a, il23, ifng, tnfa, tgfb, cxcl1, baff, april*, and *gmcsf*; the transcription factors studied were *foxp3* and *rorgt*; epithelial barrier and IgA-related genes were *zo-1, occludin*, and *pIgR*; mucin genes *muc1, muc2, muc3, muc4, muc6*, and *muc13*. Primer sequences and PCR conditions are available upon request.

#### Determination of Total IgA in Stools

On days 7, 14, and 21 of the experimental protocol the level of total IgA in stools was measured by ELISA according to the technique described by BD Pharmigen. Briefly, Maxisorp Nunc plates were coated overnight with purified rat anti-mouse IgA (BD 556969), washed with PBS containing 0.05% v/v Tween 20 (PBS-T), and blocked with FBS 10% v/v in PBS. Plates were incubated for 2 h at room temperature with purified mouse IgA kappa (BD 553476) or fecal samples. Plates were revealed using biotin rat anti-mouse IgA (BD 556978), streptavidin horseradish peroxidase (BD 554066), and trimethylbenzidine (TMB substrate reagent set BD OptEIA 555214). All determinations were performed in triplicate.

#### Fecal Microbiota Evaluation

##### Qualitative Analysis by PCR-DGGE

The experiments were performed as described previously (42). Briefly, HDA1 and HDA2-GC primers were used to assess microbial diversity in each sample. PCR products were separated in 8% polyacrylamide gels with a range of 30–50% denaturing gradient (100% denaturant consisted of 7 M urea and 40% deionized formamide) cast with Bio-Rad’s Model 475 gradient delivery system (BioRad, Hercules, CA, USA). The electrophoresis was performed in TAE 0.5× buffer for 5 h at a constant electric current of 125 mA and a temperature of 60°C with the DCode Mutation Detection System (Bio-Rad, Hercules, CA, USA). Clustering analysis was performed using the UPGMA (unweighted pair group method with arithmetic mean clustering algorithm) to calculate the dendrograms.

##### Microbiota Population Analysis in Feces by q-PCR

Microbiota population analysis in feces was performed on the day 21 of the experience as described previously (42). Briefly, DNA was extracted using the NucleoSpin Soil Genomic DNA isolation kit (Macherey-Nagel), and the quantification of bacterial populations was carried out using primers synthesized by Biomers (France). The populations evaluated were: *Firmicutes, Lactobacillus* spp., *Lactobacillus murinus, Lactobacillus acidophilus* group, *Clostridium leptum* group, *Clostridium perfringens, Clostridium coccoides* group, *Faecalibacterium prausnitzii, segmented filamentous bacteria, Enterobacteriaceae, E. coli, Bacteroidetes, Bacteroides fragilis* group, *Prevotella* group, *Akkermansia muciniphila*, and *Bifidobacterium* (42). PCR reactions were performed on a CHROMO 4™ System (Bio-Rad) using Maxima SYBR Green/ROX qPCR Master Mix (Fermentas, France).

### Statistical Analysis

Statistical comparisons for significant differences were performed according to the Student’s *t*-test. *p* Value <0.05 was considered as statistically significant.

## Results

### Strain Identification and Safety Assessment

In this study, 15 bacteria were identified to species level by 16S rDNA gene sequencing (43) and species-specific primers based on superoxide dismutase (*sodA*) gene (33). Ten strains out of 15 were identified as *E. faecium*, 4 as *E. durans*, and 1 as *Enterococcus hirae*.

Once identified, safety aspects were evaluated. None of the four *E. durans* strains were positive for any virulence or vancomycin resistance genes and the same results were obtained for *E. hirae* (Table 1). In contrast, all 10 food and animal *E. faecium* isolates were positive for *acm* (Table 1). It is important to notice that neither *E. faecium* nor *E. durans* strains was α-hemolytic. Regarding antibiotic resistance, 90% of *E. faecium* strains and *E. hirae* were resistant to two or more antibiotics, while only 25% of *E. durans* strains showed this profile (data not shown).

Considering the described results, three *E. durans* strains were selected to perform *in vitro* studies on PBMC and Caco-2 cell line.

### *E. durans* Strains Stimulates PBMC and Modulates Proinflammatory Molecules on Caco-2 Cells

The three strains of *E. durans* (EP1, EP2, and EP3) chosen after *in vitro* safety evaluation were cocultured with human PBMC and Caco-2 cells. Secreted cytokines and gene expression was assessed.

On PBMC, quantification of secreted cytokines in supernatant showed a similar pattern for the three strains under study (Table 2). All of them increased IL-1β, IL-6, IL-12p70, IFN-γ, and TNF-α as well as IL-10. However, EP1 induced the lowest TNF-α/IL-10, IL-1β/IL-10, and IL-12/IL-10 ratios (Table 2), suggesting that EP1 is a better anti-inflammatory candidate (44).

**Table 2:** **Cytokine production by PBMC and selected cytokine ratio after 48-h exposure to *E. durans* strains**.

	EP1	EP2	EP3	Control PBMC
**Cytokine (pg/mL)**				
IL-1β	1,226 ± 13^b^	2,025 ± 25^b^	1,642 ± 180^b^	25 ± 12^a^
IL-6	3,762 ± 256^b^	3,362 ± 210^b^	3,785 ± 169^b^	66 ± 19^a^
IL-8	3,368 ± 425^a^	4,585 ± 769^a^	3,685 ± 225^a^	4,153 ± 211^a^
TNF-α	7,315 ± 3,861^b^	24,508 ± 3,354^c^	20,261 ± 5,499^c^	228 ± 43^a^
IFN-γ	118 ± 16^b^	66 ± 9^b^	109 ± 4^b^	8 ± 2^a^
IL12p70	871 ± 30^b^	1,011 ± 57^b^	1,183 ± 27^b^	403 ± 162^a^
IL-10	1,171 ± 41^b^	1,345 ± 36^b^	1,485 ± 351^b^	6 ± 2^a^
**Cytokine ratio**				
IL-1β/IL-10	1.05	1.50	1.11	4.17
IL-12/IL-10	0.74	0.75	0.80	67.17
TNF-α/IL-10	6.25	18.22	13.64	38.00

*Means with the same letter for each parameter are not significantly different*.

Stimulation assays performed with confluent Caco-2 cells also revealed differences between strains. EP2 increased expression of IL-6 (*p* < 0.05) but did not affect IL-1β and IL-8 expression. On the other hand, EP1 decreased expression of IL-1β, IL-6, and IL-8 (*p* < 0.05), while EP3 did not affect expression of any tested genes (data not shown).

Considering all the obtained results, the strain *E. durans* EP1 was selected to evaluate probiotic properties and its *in vivo* immunomodulatory effect in mice.

### EP1 Resists Gastrointestinal Conditions without Modifying Its Adhesion Capacity and Also Inhibits Growth of Gram-Positive and -Negative Pathogens

The ability of *E. durans* EP1 to survive to the simulated gastrointestinal conditions was assessed. Viability was not affected by gastric step. On the contrary, the critical step was the intestinal passage which lowered viability significantly around one logarithmic unit.

**Figure 1:**
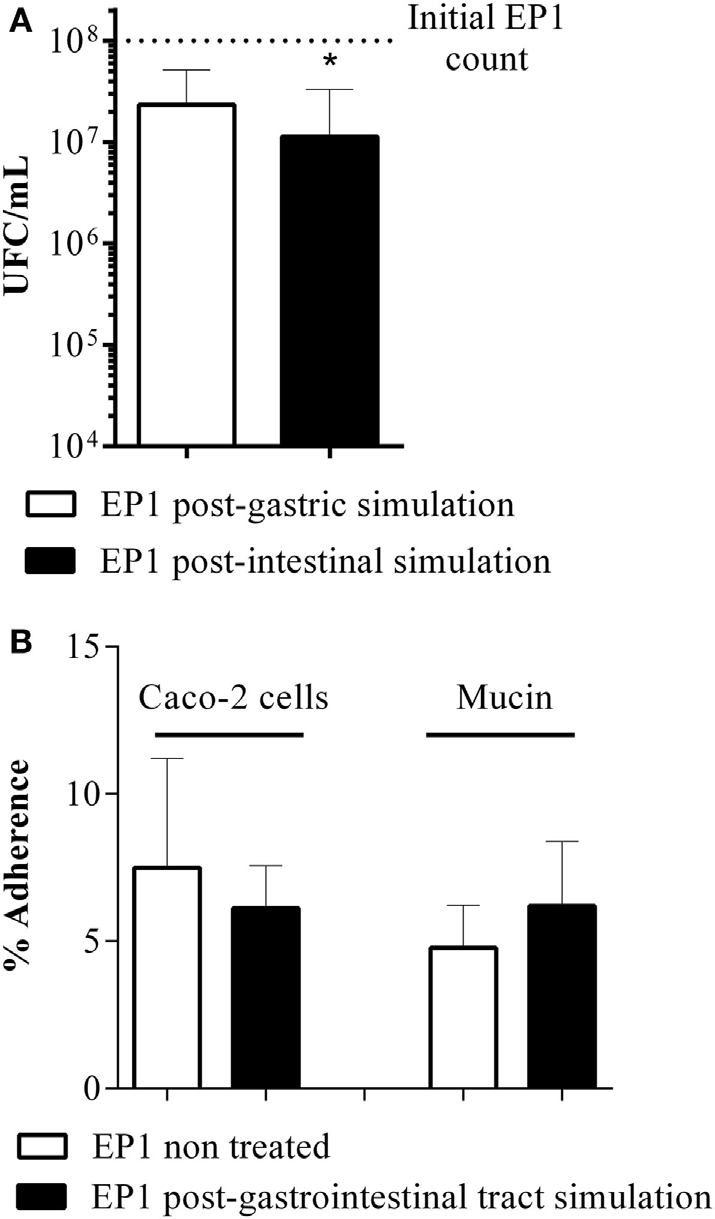
**Probiotic properties of EP1**. **(A)** Resistance to simulated gastrointestinal conditions. **(B)** Percentage of adhesion to Caco-2 cell line and gastric mucin of EP1 cells and EP1 cells post-gastrointestinal tract simulation. Results are expressed as mean ± SD and are representative of at least three independent experiments (*p < 0.05).

On the other hand, *E. durans* EP1 presented a moderate adhesion capacity (around 6–8%) to both porcine gastric mucin and Caco-2 cells, which was not affected by the passage through the gastrointestinal tract simulation (Figure 1).

Moreover, *E. durans* EP1 is able to inhibit the growth of pathogens *in vitro*. We observed that EP1 exerts a powerful inhibitory effect on *S. aureus* and *L. monocytogenes* and a moderated effect on *E. coli, S. flexneri, S. enterica*, and *P. aeruginosa* (Table 3). On the contrary, *B. cereus* and *E. faecalis* were slightly or not inhibited.

**Table 3:** **Growth inhibition of bacterial pathogens by EP1**.

Gram-positive pathogens	*R* (mm)	Gram-negative pathogens	*R* (mm)
*Shigella flexneri* ATCC 9199	7	*Listeria monocytogenes* ATCC 7644	15
*Pseudomonas. aeruginosa* ATCC 15442	9	*S. aureus* ATCC 6538	12
*Salmonella enterica* CIDCA 101	5	*Enterococcus faecalis* ATCC 29212	3
EHEC EDL933	5	*Bacillus cereus* ATCC 10876	<1

*EHEC, enterohaemorragic Escherichia coli; R, width of clear zone around colony*.

*No inhibition (width of the clear zone around colony <2 mm); low inhibition capacity (width of the clear zone around colony between 2 and 5 mm); high inhibition capacity (width of clear zone around colony >6 mm)*.

### *In Vivo* Studies

#### *Enterococcus durans* EP1 Shows No Deleterious Effect on Swiss Mice

**Figure 2:**
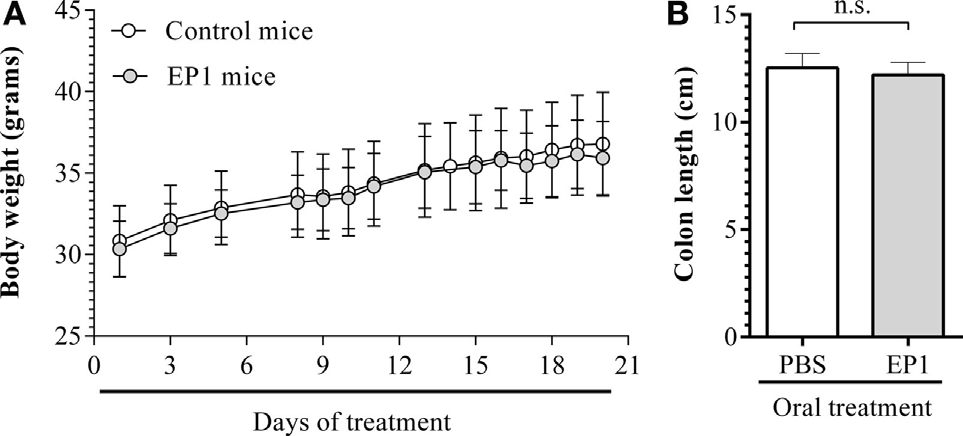
***Enterococcus durans* EP1 administration for 21 days had no deleterious effect on Swiss mice**. **(A)** Body weight gain of treated (EP1) and control mice. **(B)** Colon length at the end of the experimental protocol, results expressed as mean ± SD.

There were no differences in food and water intake between mice that received 100 µL of a 10^9^ CFU mL^−1^ suspension of *E. durans* EP1 (EP1 group) and mice receiving 100 µL of PBS (control group) daily for 21 days (data not shown). Moreover, no differences in body weight were observed between groups (Figure 2A). EP1 group did not show any signs of pain, lethargy, dehydration, or diarrhea during treatment. In accordance with these observations, no signs of inflammation or damage were observed in any organ during necropsy, and no significant differences in colon’s length (45) between EP1 and control mice were observed (12.4 ± 0.5 vs 12.6 ± 0.8 cm; Figure 2B). Finally, the histological evaluation of ileum and colon sections of EP1 group did not show any signs of inflammation, such as edema, erosion/ulceration, crypt loss, or infiltration of mono- and polymorphonuclear cells (data not shown). On the other hand, no bacterial growth was observed in any of the used cultured media, thus no translocation of microorganisms to blood, spleen, or liver was observed.

#### *Enterococcus durans* EP1 Administration Increases sIgA in Feces

**Figure 3:**
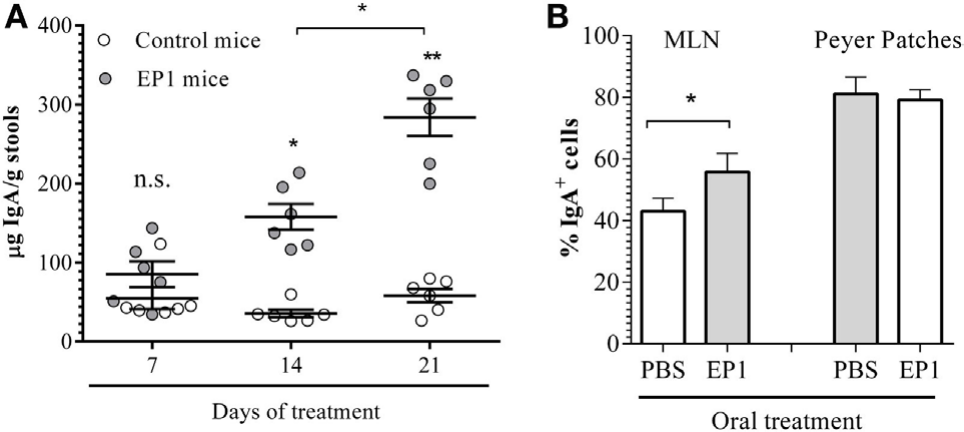
**Impact of EP1 administration on sIgA**. **(A)** IgA quantification from fecal samples taken on day 7, 14, or 21 from control mice and EP1-treated mice (EP1). **(B)** Percentage of IgA+ cells in mesenteric lymph node and Peyer’s Patches after 7 days of EP1 administration. Results are expressed as mean ± SD. No significant differences (n.s.); *p < 0.01; **p < 0.001.

Total sIgA concentration was increased in mice stools after treatment with EP1. SIgA increases progressively throughout the treatment (Figure 3A). The average increment of secreted sIgA after 1 week of probiotic administration was of 0.7 times meanwhile increments of 3.2 and 5.75 relative to control group were observed after 2 and 3 weeks, respectively (Figure 3A).

Even though the increment of secreted sIgA was not significantly increased after 7 days of EP1 administration, the number of IgA+ cells was significantly higher in MLN of treated mice than in control mice (Figure 3B). In contrast, this was not observed in Peyer’s Patches (Figure 3B).

#### *Enterococcus durans* EP1 Downregulates the Expression of Proinflammatory Molecules and Mucins in Ileum and Modifies Its Reactivity to LPS

**Figure 4:**
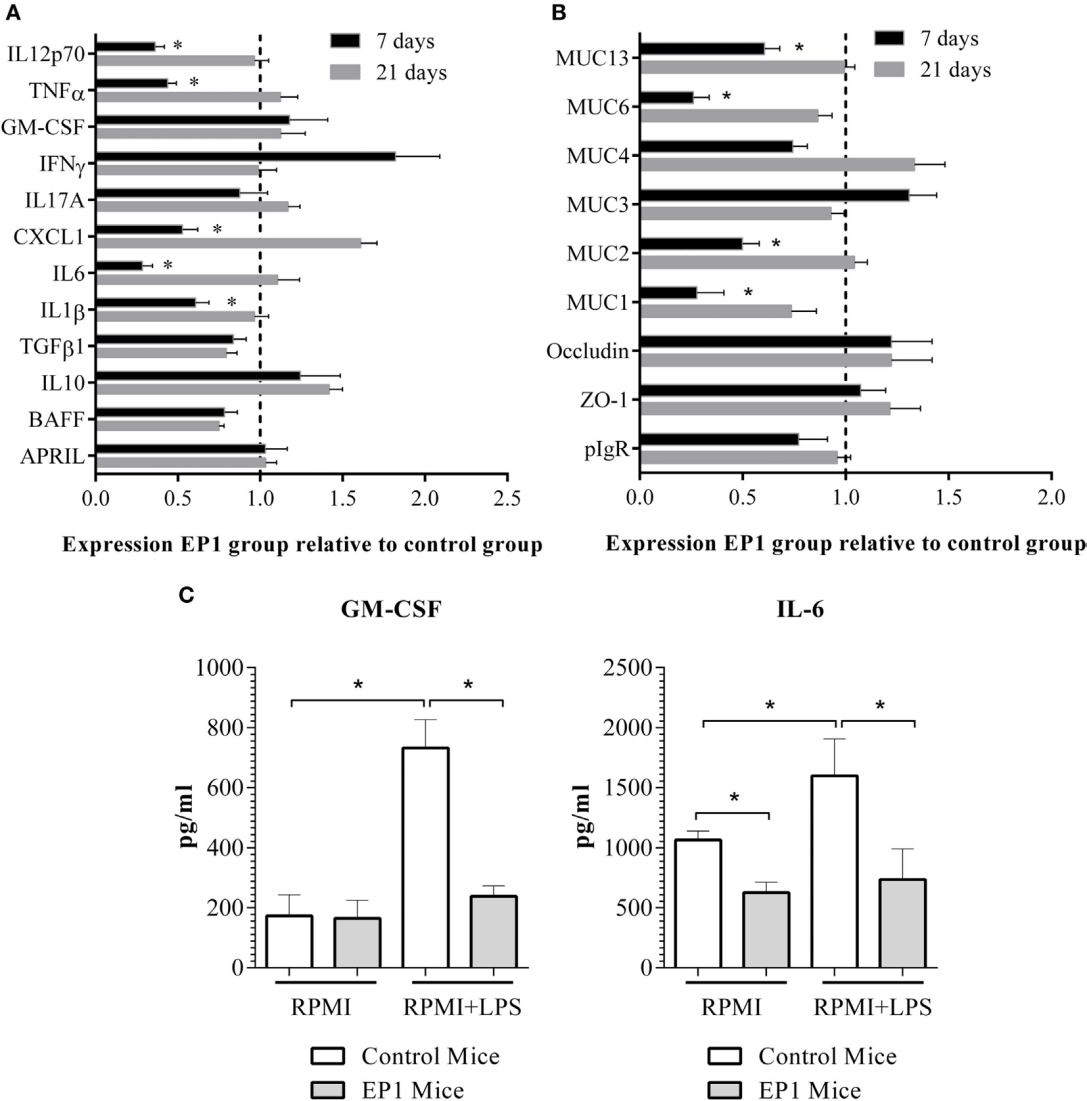
**Effect of EP1 administration on ileum**. **(A)** Expression of cytokines and chemokines. **(B)** Expression of genes related with intestinal epithelial barrier function. **(C)** Cytokines in supernatants of 21 days treated ileum explants cultured for 48 h in the absence or presence of LPS. Results are expressed as mean ± SD (*p < 0.01).

The effect of *E. durans* EP1 administration on ileum gene expression was assessed after 7 and 21 days of treatment. The analysis of cytokines’ and chemokines’ expression showed a decrease in mRNA amounts of the proinflammatory molecules *il6, il1b, il12p70*, and *tnf-a* after 7 days, but no differences in expression after 21 days of administration (Figure 4A) were observed. Interestingly, mucin genes were also downregulated at the first week of probiotic treatment but after a longer administration period the expression of these molecules returned to the levels observed in control mice (Figure 4B).

Thereupon, we decided to assess ileum reactivity by culturing tissue sections in presence or absence of the proinflammatory stimuli LPS. We observed that ileum sections from 21 days EP1 treated mice without any stimulation secreted lower quantities of IL-6 (Figure 4C) and higher amounts of IL-10 (165 ± 57 vs 25 ± 12 pg/mL) than control mice. Moreover, LPS stimulation induced lower amounts of GM-CSF and IL-6 in EP1 group (Figure 4C). Levels of IL-4, TNF-α, IL-17A, and IFN-γ were not modified after stimulation in either group (data not shown).

#### *E. durans* Reduces Proinflammatory Cytokines Levels and Mucins Expression in Colon and Increases IL-10 Secretion in Response to LPS Stimulation

**Figure 5:**
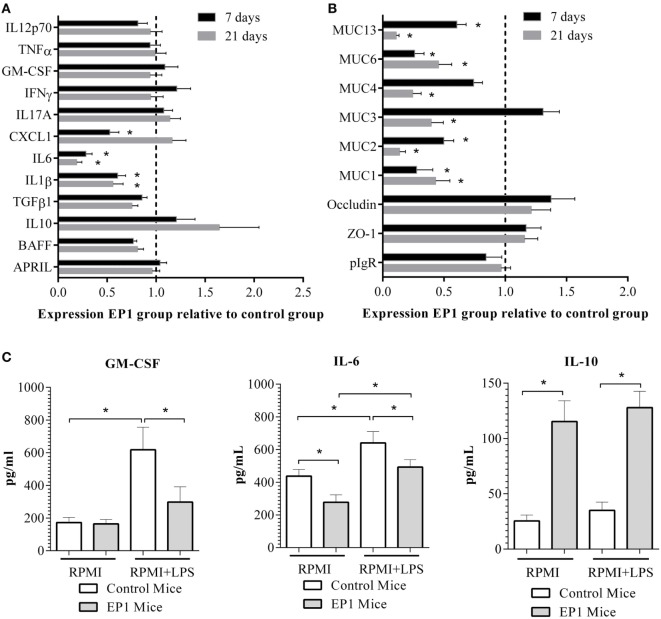
**Effect of EP1 administration in colon**. **(A)** Expression of cytokines and chemokines. **(B)** Expression of genes related with intestinal epithelial barrier function. **(C)** Cytokines in supernatants of 21 days treated colon explants cultured for 48 h in the absence or presence of LPS. Results are expressed as mean ± SD (*p < 0.01).

EP1 consumption decreased *il6, il1b*, and *cxcl*-1 expression after 7 days, and this downregulation was persistent for the first two genes after 21 days of probiotic treatment (Figure 5A). On the other hand, mucin-encoding genes showed a decreased expression after 1 and 3 weeks of EP1 administration (Figure 5B). Afterward, reactivity to LPS was assessed. As shown in Figure 5C, not stimulated colon explants from 21 days EP1-treated mice secreted lower amounts of IL6 and higher quantities of IL10 than control mice. In accordance, the proinflammatory stimuli LPS produced a lower increment of GM-CSF and IL-6 in EP1 group and a higher secretion of IL-10 (Figure 5C). As it was observed in ileum explants, levels of IL-4, TNF-α, IL-17A, and IFN-γ showed no changes after stimulation (data not shown).

#### *E. durans* Decreases the Expression of Proinflammatory Cytokines in MLN and PP

**Figure 6:**
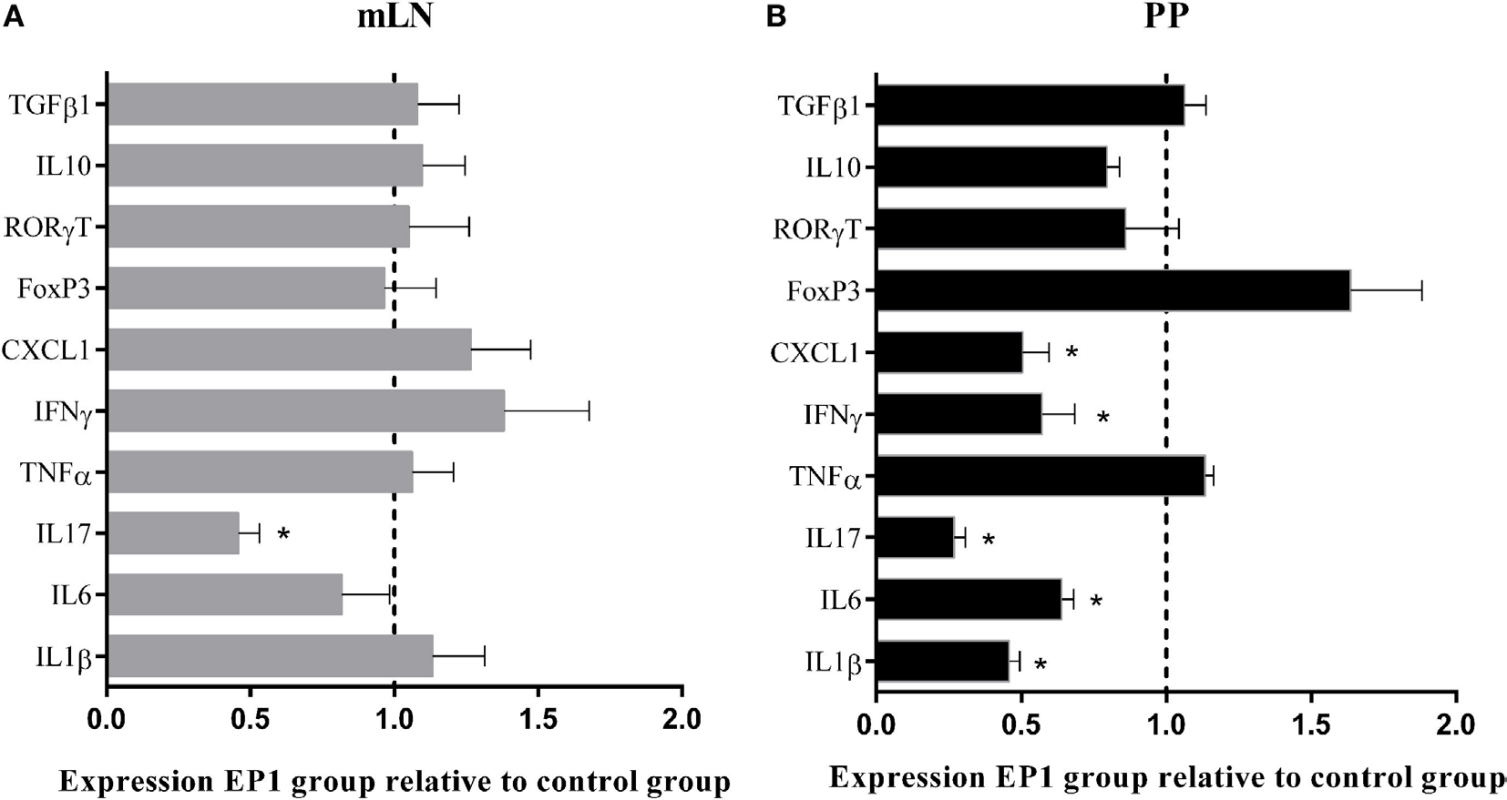
**Gene expression in mucosal induction sites after 21 days of EP1 administration, (A) mesenteric lymph nodes (B) Peyer’s patches**. Results are expressed as mean ± SD (*p < 0.05).

Since immune modulation was observed in ileum and colon from mice treated with EP1, we decided to evaluate the impact of probiotic administration in the induction sites MLN and PP. We observed that treatment with EP1 during 21 days decreased the expression of the proinflammatory molecules *il1b, il6, ifng*, and *cxcl-1* only in PP while *il-17* was reduced in both MLN and PP (Figure 6). This last result is particularly interesting since EP1 not only decreased the expression of Th1 related genes but also Th17-related molecules.

#### EP1 Administration Increases *F. prausnitzii* Amount in Mice Stools

**Figure 7:**
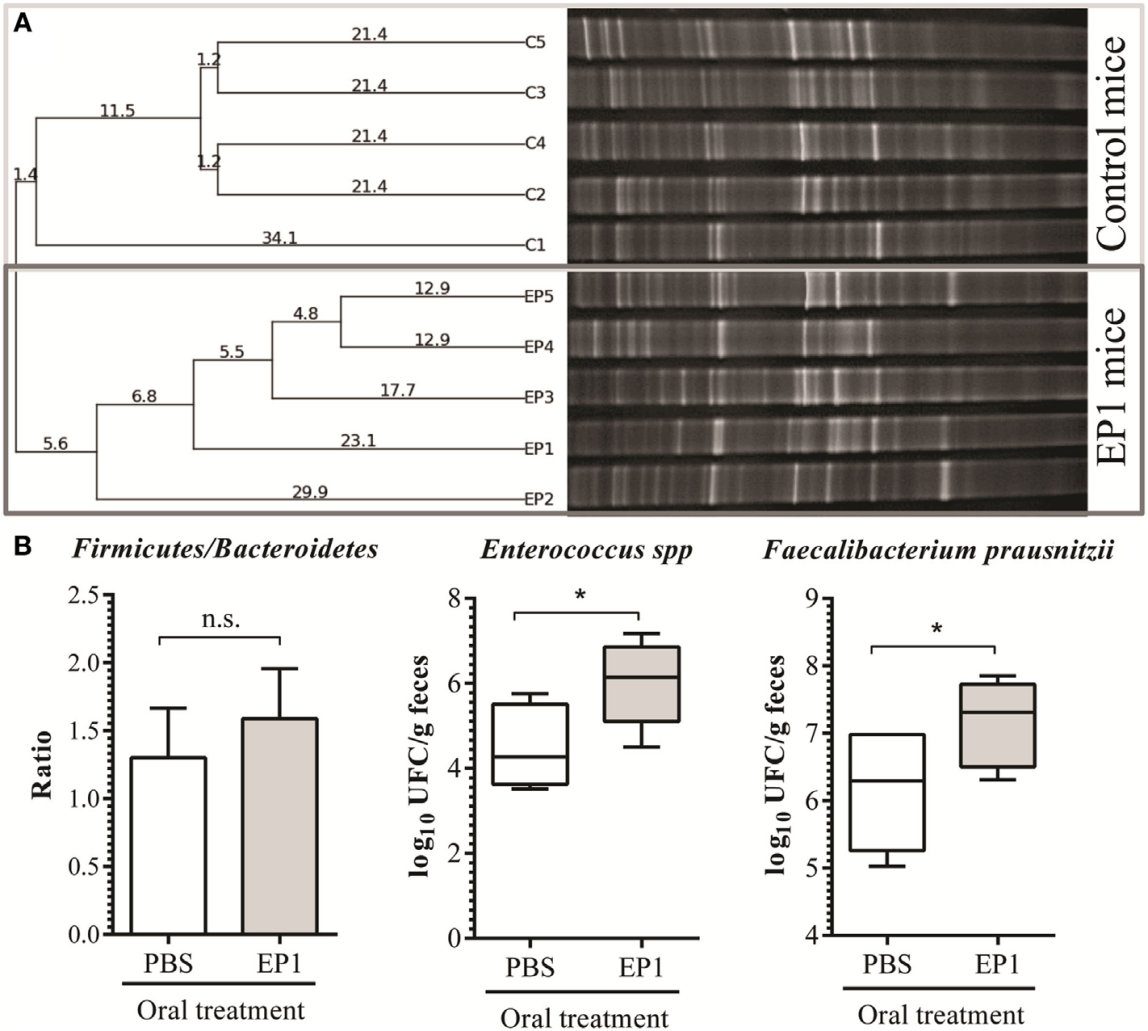
**Impact of treatment with EP1 for 21 days on fecal microbiota**. **(A)** Total bacteria DGGE profiles and dendrogram of five mice from control group (C1–C5) and five from EP group (lanes EP1–EP5). Clustering analysis was performed using the UPGMA linkage. **(B)** qPCR quantification results for *Firmicutes/Bacteroidetes* ratio, and total count for *Enterococcus* spp and *Faecalibacterium prausnitzii* (*p < 0.05).

The impact of EP1 administration in fecal microbiota was assessed by qualitative (PCR-DGGE) and quantitative (qPCR) methods. The number of PCR-DGGE amplified bands can be related with microbial diversity. No significant differences were observed between control and EP1 mice (32 ± 3 vs 34 ± 4) indicating that the probiotic does not alter bacterial diversity in healthy conditions. However, the cluster analysis based on the Pearson product-moment correlation coefficient and UPGMA linkage allowed differentiation of the experimental groups in two clusters which indicates changes in the microbial community composition due to probiotic administration (Figure 7A).

The parameters initially evaluated by qPCR were total bacterial load, *Firmicutes/Bacteroidetes* ratio, and *Enterobacteriaceae* quantities, particularly *E. coli*, since changes in these parameters are associated with non-healthy microbiota (46). In correlation with the results exposed previously in this work, no changes in the mentioned parameters were observed in mice treated with EP1. As expected, the quantitative methods revealed an increment in *Enterococcus* population (Figure 7B). Interestingly, an increment in the Gram-positive butyrate-producing bacterium *F. prausnitzii* belonging to *Clostridium* cluster IV, was detected in stools from EP1-treated mice (Figure 7B). No significant changes were observed in any other quantified population, not even in *A. muciniphila* which was perhaps expected to be affected due to the decrease in mucin expression in EP1-treated mice compared with controls.

## Discussion

*Enterococcus* strains have been used as long time as effective probiotics but this bacterial group can also harbor pathogenic strains. In this context, it is indispensable to analyze the presence of virulence factors and antibiotic resistances. Generally, the frequency of pathogenic strains is higher in *E. faecalis* and *E. faecium*; however, some authors have retrieved occasionally *E. durans* isolates from foods or healthy children stools possessing virulence factors (8, 47). The strain that we selected, *E. durans* EP1, is in agreement with the requirements established by the EFSA (11). Furthermore, no deleterious effect was observed in mice that received EP1 for 21 days.

Adhesion to gastrointestinal mucus and epithelial cells has an important role in the probiotic effect and can be related to the immunomodulation properties (48). EP1 presents a moderate adhesion to mucin and Caco-2 cells (around 6–8%) such as described for some *Bifidobacteria* and *Lactobacillus* strains (48–50). Interestingly, this property is not affected after gastrointestinal tract passage simulation.

In order to screen the immunomodulatory activity of the selected *E. durans* strains, we used PBMC from healthy donors and the results obtained were strain dependent. Considering the pro-inflammatory/anti-inflammatory cytokines ratio, the lower values were obtained for EP1 suggesting that this strain has better anti-inflammatory potentiality. In this sense, other authors have demonstrated the correlation between this ratio and the *in vivo* anti-inflammatory expected effect (42, 44, 51). In our *in vivo* study, we also corroborate the predictive power of these ratios.

Most of the studies performed to evaluate the effects of probiotics on the immune system use animal disease models. Of equal or higher interest is the study of immune modulation in healthy individuals as the knowledge acquired can be used to prevent specific pathologies or disease development (52–54). To gain insight in how *E. durans* EP1 modulates the immune system, healthy mice were treated orally with the strain for 21 days. After 1 week of probiotic treatment, expression of proinflammatory cytokines was downregulated in both ileum and colon. On the 21st day, expression levels of all the evaluated genes in ileum returned to those found in control mice meanwhile in colon proinflammatory cytokines were still downregulated. The differences in expression observed between tissues could be related to the presence of EP1 in each section of the gut or with modifications in the quantity and composition of the local microbiota as well as differences in the thickness of the mucus layer that may affect bacterial interaction with host’s cells (52, 55, 56). Similar results were obtained by Smelt et al. (52), who evaluated the impact of several *Lactobacillus* and observed distinct changes in *lamina propia* of small and large intestine of healthy mice.

Despite of the differences in mRNA quantities in ileum and colon after 21 days of treatment, a lower basal secretion of IL-6 and a higher of IL-10 were observed in tissue explants from EP1-treated mice. Moreover, EP1 showed significant anti-inflammatory effect, as evidenced by the suppression of LPS-induced IL-6 and GM-CSF levels in both tissues explants and upregulation of IL-10 amounts in colon. In accordance with these results, several proinflammatory cytokine genes were downregulated in PP and IL17 in MLN at day 21. The described findings suggest that this strain could have a positive effect on intestinal inflammation (44, 57). Even though the overall anti-inflammatory phenotype of EP1 is similar to that of *Lactobacillus kefiri* CIDCA 8348 (same cytokine ratio after PBMC stimulation and similar anti-inflammatory response of intestinal explants to LPS), there are differences in the mucosal response *in vivo*. *E. durans* reduces IL-6 expression in colon which appears be concomitant with the decrease in the expression of genes involved in mucins production. On the contrary, *L. kefiri* does not affect IL-6 expression and upregulates mucins genes [Ref. (42), see below]. These data further corroborate that mucosal immunity and homeostatic properties of probiotics are strain specific.

An important finding of the present study was that the administration of EP1 strain resulted in higher IgA content in feces. Fourteen days of probiotic treatment was sufficient to increase stools IgA levels five times, and after 21 days, the IgA level was increased near six times. Secretory IgA, the predominant immunoglobulin class in human external secretions, is a key element in the maintenance of gut microbiota homeostasis and in the protection of the mucosal epithelia against pathogens (58) and its induction has been described for other probiotic bacteria (59, 60). The production of sIgA decreases with age can lead to an increased risk of infection (61). In this context, Lefevre et al. (62) showed that the consumption of a *Bacillus subtilis* probiotic (CU1) significantly increases intestinal and salivary sIgA in seniors helping a decreased the frequency of respiratory infections.

As a result of probiotic stimulation the IgA cycle can be induced and the number of IgA^+^ cells in mucosal sites distant to the intestine can be increased (63). We observed a significant augmentation in IgA-producing cells in the MLN of mice treated with the *E. durans* EP1. These results are in accordance with the effect observed in *L. kefiri* CIDCA 8348 that occasioned an increment in IgA^+^ B cells in MLN correlating with an increase of IgA in fecal samples (42). It is interesting to note that sIgA has a dual function, (i) preventing overgrowth of the gut microbiota and (ii) also minimizing its interactions with the mucosal immune system, diminishing the host’s reaction to its resident microbes (64).

Another important feature on mucosal physiology is the mucus layer. Mucins are the main component of the mucus layer and it has been described that their production could be modified by changes in host microbiota induced by diet changes, infections, probiotic, or antibiotic treatments (65–67). EP1 administration decreased the expression of mucins in both ileum and colon. The observed downregulation could be explained by a direct effect of *E. durans* EP1 on mucosa or as a result of changes induced in mice microbiota after probiotic treatment. The increment in *Faecalibacterium prausnitzii* is of interest since it has been described that it can modulate the effects of other bacteria on goblet cells and thus decrease mucus production and mucin glycosylation (68).

Even though it has been proposed that thinning of the mucus layer may increase contact between epithelial cells and bacteria present in the microbiota, augmenting the inflammatory tone of the intestine, this effect was not observed in this study. The fact that sIgA is increased in EP1 mice may be related with this observation since it has been described that microbiota are linked with sIgA to control intestinal homeostasis and that spatial segregation of pathobionts from the intestinal wall occurs as a result of intraluminal agglutination in an extracellular matrix consisting of sIgA, polymeric immunoglobulin receptor, and epithelial cadherin (E-cadherin) proteins (64, 69). Moreover, increased expression of mucins is often associated with invasive bacteria and inflammatory conditions (70–72). In accordance with our results, Levkut et al. (73) observed that *E. faecium* administration to broilers induced a decrease in mucus layer density. Interestingly, the probiotic treatment exerted a protective effect when chickens were challenged with *Salmonella*.

It is known that an active dialog exists between the commensal microorganisms and the host mucosal immune system (63, 74). Probiotics may help to maintain immune functions and mucosal homeostasis either by direct interaction with the host or indirectly by re-equilibrating or modulating the gut microbiota (75, 76). *Enterococcus* species are known to be great antimicrobial producers (17, 77), a good example was shown by Nami et al. (78) using the *E. durans* strain 6HL isolated from the vagina of heathy women. In this work, we observed that *E. durans* EP1 produces antimicrobials substances since it inhibits growth of several pathogens *in vitro*. These secreted substances can be implicated in microbiota modulation. Analysis of mice microbiota demonstrated that EP1 administration increased *F. prausnitzii*, while all other tested populations remained unchanged. Unexpected was the preservation of *A. muciniphila* count since this bacteria is a mucin-degrading member of the intestinal microbiota (79) and could have been affected by the decrease in mucin gene expression.

*Faecalibacterium prausnitzii* is one of the most abundant bacteria in the human gut ecosystem, and it is an important supplier of butyrate to the colonic epithelium (80). We hypothesized that *F. prausnitzii* resists better the antimicrobials molecules produced by EP1 than the other members of *C. leptum* group (cluster IV). Interesting, *F. prausnitzii*, a member of the human microbiota “core” is very important for intestinal homeostasis maintenance and is known to elicit strong anti-inflammatory responses. In fact, *F. prausnitzii* has been associated with longer remission periods in Crohn’s disease patients (80). Moreover, *F. prausnitzii* increases Treg cells counts in which suggests their therapeutic potential for the treatment of diseases associated with loss of tolerance (81). We presume that the increment of *F. prausnitzii* in mice treated with *E. durans* EP1 is involved in the anti-inflammatory effects observed in mice mucosa.

## Conclusion

*Enterococcus durans* strain EP1, selected by the evaluation of pro-inflammatory/anti-inflammatory cytokines ratio in PBMC, has no virulence factors and has no deleterious effect on mice. We demonstrated that this strain is a strong sIgA inducer and possess anti-inflammatory properties, downregulating the expression of pro-inflammatory cytokines in the intestinal mucosa and inducing the secretion of IL-10. EP1 modulates gut microbiota increasing the anti-inflammatory bacteria *F. prausnitzii* that could be implicated also in the observed anti-inflammatory responses.

Thus, *E. durans* EP1 is not only a good candidate to increases *F. prausnitzii* in elderly population or other dysbiotic situations but also for gut inflammatory disorders therapy. We will continue the study of this strain in a mice model of inflammation in our laboratory.

## Author Contributions

PC contributed to the conception and design of the work, the acquisition, analysis and interpretation of the data, discussion, and writing of the manuscript. SR contributed to the conception and design of the work and to the acquisition of data. CJ and AE worked on the gene expression analysis and microbiota studies. MS helped analysing data and writing of the manuscript. MU coordinated the work, analysis of results, discussion, and writing of the manuscript. All the authors have approved the final version of the manuscript.

## Conflict of Interest Statement

The authors declare that the research was conducted in the absence of any commercial or financial relationships that could be construed as a potential conflict of interest. The reviewer SS and handling Editor declared their shared affiliation, and the handling Editor states that the process nevertheless met the standards of a fair and objective review.

## Abbreviations

CXCL-1, chemokine (C-X-C motif) ligand 1; GM-CSF, granulocyte macrophage colony-stimulating factor; IFN, interferon; IL, interleukin; MLN, mesenteric lymph nodes; PBMC, peripheral blood mononuclear cell; PP, Peyer’s patches; TNF, tumor necrosis factor.
